# Defective glucocorticoid receptor signaling and keratinocyte-autonomous defects contribute to skin phenotype of mouse embryos lacking the Hsp90 co-chaperone p23

**DOI:** 10.1371/journal.pone.0180035

**Published:** 2017-06-26

**Authors:** Marta Madon-Simon, Iwona Grad, Pilar Bayo, Paloma Pérez, Didier Picard

**Affiliations:** 1 Département de Biologie Cellulaire, Université de Genève, Sciences III, 30 quai Ernest-Ansermet, Genève 4, Switzerland; 2 Instituto de Biomedicina de Valencia—Consejo Superior de Investigaciones Científicas (IBV-CSIC), Jaime Roig 11, Valencia, Spain; University of Alabama at Birmingham, UNITED STATES

## Abstract

p23 is a small acidic protein with intrinsic molecular chaperone activity. It is best known as a co-chaperone of the major cytosolic molecular chaperone Hsp90. p23 binds the N-terminus of Hsp90 and stabilizes the ATP-bound and N-terminally closed Hsp90 dimer. It is in this configuration that many Hsp90 clients are most stably bound. Considering the important role of p23 in the Hsp90 cycle, it came as a surprise that it is not absolutely essential for viability in the budding yeast or for mouse development. Mice without p23 develop quite normally until birth and then all die perinatally because of immature lungs. The only other apparent phenotype of late stage embryos and newborns is a skin defect, which we have further characterized here. We found that skin differentiation is impaired, and that both apoptosis and cell proliferation are augmented in the absence of p23; the consequences are a severe thinning of the stratum corneum and reduced numbers of hair follicles. The altered differentiation, spontaneous apoptosis and proliferation are all mimicked by isolated primary keratinocytes indicating that they do require p23 functions in a cell-autonomous fashion. Since the phenotype of p23-null embryos is strikingly similar to that of embryos lacking the glucocorticoid receptor, a paradigmatic Hsp90-p23 client protein, we investigated glucocorticoid signaling. We discovered that it is impaired *in vivo* and for some aspects in isolated keratinocytes. Our results suggest that part of the phenotype of p23-null embryos can be explained by an impact on this particular Hsp90 client, but do not exclude that p23 by itself or in association with Hsp90 affects skin development and homeostasis through yet other pathways.

## Introduction

p23, whose official name is Ptges3 for rather arcane historical reasons, is a small, acidic protein that was initially discovered as an essential component of the Hsp90 chaperone machinery required for correct assembly of steroid receptor complexes [1,2]. Since then many other Hsp90 clients were found to rely on the contribution of p23 as an Hsp90 co-chaperone (for comprehensive overview, see https://www.picard.ch/downloads/p23facts.pdf). The direct binding of p23 to the N-terminal domain of Hsp90 is ATP-dependent [3,1] and results from the ATP-induced N-terminal dimerization of Hsp90 [4,5]. p23 inhibits both the basal and substrate-stimulated ATPase activity of Hsp90 resulting in the stabilization of the nucleotide-bound closed state of Hsp90 [6–8]. Apart from being an important regulator of the Hsp90 chaperoning cycle [9,8], p23 has its own Hsp90-independent chaperone activity, which can prevent protein aggregation and maintain proteins in a folding-competent state [10–12]. At the cellular level, p23 is mostly cytoplasmic, but can also be found in the nucleus [13–15]. In the nucleus, in addition to acting as a co-chaperone for Hsp90 present in the nucleus p23 deploys its chaperone activity to promote the dynamic binding and dissociation of DNA- and chromatin-binding complexes [16–19].

The Hsp90 co-chaperone p23 is found in almost all eukaryotes [20]. In the mouse, it is ubiquitously expressed with the exception of striated muscle, where it is replaced by the related co-chaperone Aarsd1 [16,21]. The crucial role of p23 in the mouse has been demonstrated by our group [22] and others [23,24]. *p23* gene disruption mice die at birth, most likely because of the immaturity of their lungs; in addition, late stage embryos and newborns display a significant underdevelopment of the skin; notably, the stratum corneum is much thinner and the skin barrier function is defective [22]. The tissue selectivity of this phenotype was surprising in view of the expected house-keeping functions of p23 and Hsp90. The phenotype is strikingly reminiscent of what has been reported for the knockout of the gene for the glucocorticoid receptor (GR) [25–28], a paradigmatic Hsp90 client protein (reviewed in refs. [29,30]). GR is an important regulator of skin development and homeostasis and responds to both systemic sources of glucocorticoids and glucocorticoids produced locally from cholesterol in the skin itself [31–34]. Upon first describing the skin phenotype [22], we therefore explored the connection with GR. We found that mouse embryonic fibroblasts derived from *p23* null embryos showed abnormalities in GR functions: although they could still respond to glucocorticoids, it took longer or higher glucocorticoid concentrations [22]. The findings both with the lung and the skin were compatible with the speculation that GR may be the main p23 target, at least during the late stages of embryonic development.

While the skin phenotype of GR mutant mice has been extensively studied [26–28,35,36], our own characterization of the skin phenotype of p23 null mice had been relatively limited and no additional study has been reported since then. Here we have revisited the skin phenotype of *p23* null mice in more detail and further investigated the p23-GR connection.

## Results

### Effects on proliferation, differentiation, and apoptosis in the skin

**Fig 1:**
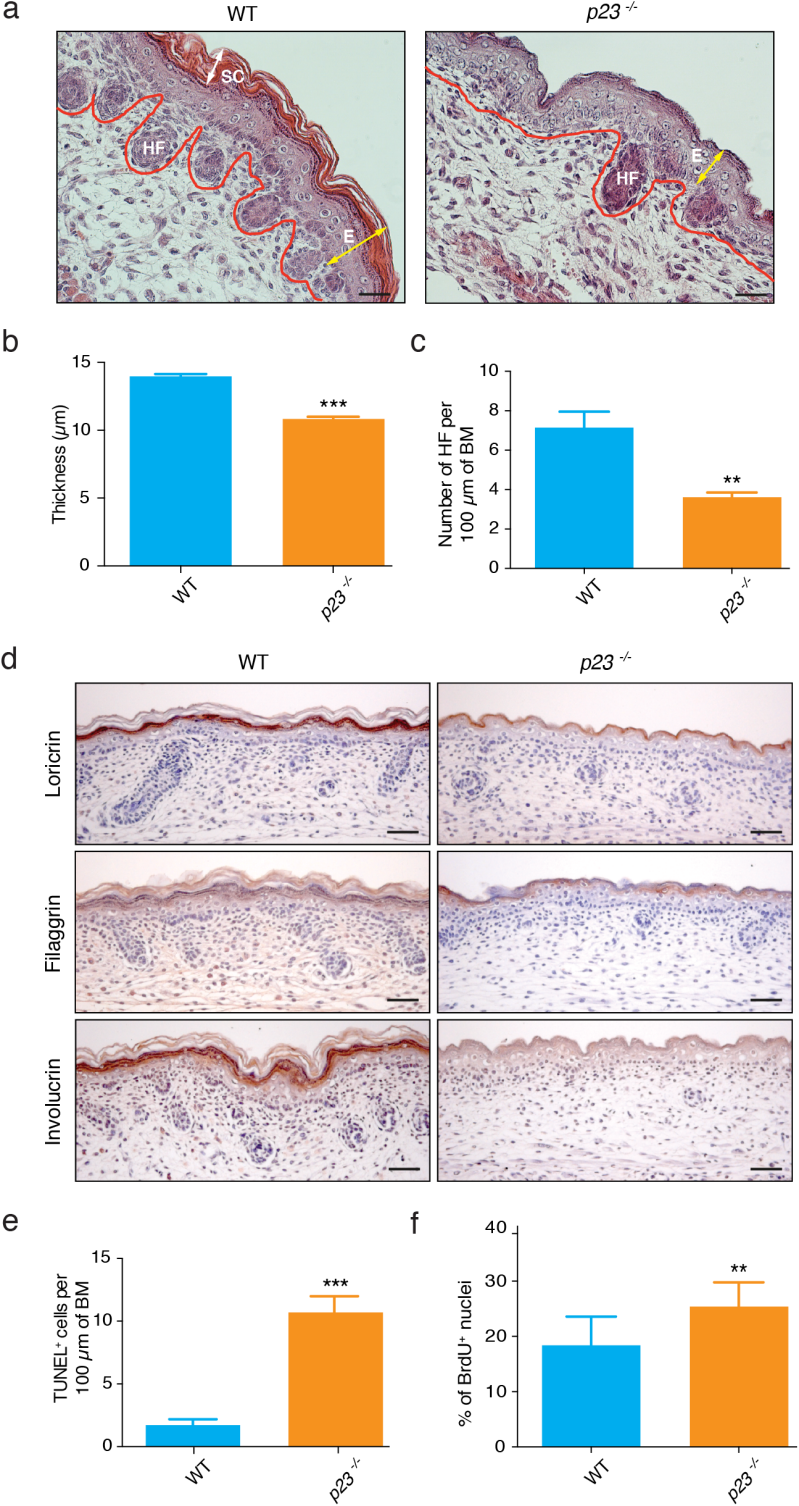
Lack of p23 results in decreased epidermal thickness, delayed differentiation and increased rates of proliferation and apoptosis in E18.5 skin. (**a**) Representative sections stained with H&E showing epidermal skin (delimited by red line) thinning with almost absent stratum corneum in embryonic *p23*^*-/-*^ skin sections along with lower numbers of hair follicles. E, epidermis; HF, hair follicle; SC, stratum corneum. (**b**) Quantitation of epidermal thickness and (**c**) number of hair follicle per 100 μm basal membrane (BM) in *p23*^*-/-*^ and wild-type E18.5 skin sections; significant decrease of epidermal thickness (*** p<0.001) and hair follicle numbers (** p<0.01) in *p23*^*-/-*^ skin (n: WT = 4, *p23*^*-/-*^ = 3). (**d**) Immunohistochemical staining for the differentiation markers loricrin, filaggrin and involucrin shows decrease in staining intensity in *p23*^*-/-*^ E18.5 skin sections. (**e**) TUNEL assay indicates significantly higher apoptotic rate in *p23*^*-/-*^ E18.5 skin sections compared to wild-type (*** p<0.001; n: WT = 4, *p23*^*-/-*^ = 4). (**f**) BrdU staining of nuclei indicates increased proliferation in *p23*^*-/-*^ skin sections (** p<0.01; n: WT = 7, *p23*^*-/-*^ = 7). Black bars in panels **a** and **d** indicate 50 μm.

We and others had previously reported the importance of p23 for the correct development of whole body skin in the mouse [22–24]. While these studies had provided a first characterization of the skin phenotype, a more in-depth description is needed to suggest underlying molecular defects. We set out to fill this gap by analyzing the skin of *p23*^*-/-*^ E18.5 embryos. Upon dissection, the skin devoid of p23 was found to be clearly thinner. As illustrated in Fig 1A with the sections stained with haematoxylin and eosin (H&E) and confirming our earlier observations [22], the outermost layer of the skin, the stratum corneum, is strikingly reduced. This is corroborated by the morphometric analysis (Fig 1B). In addition, the number of hair follicles in *p23*^*-/-*^ compared to wild-type skin is significantly reduced (Fig 1A and 1C). Furthermore, the immunohistochemical analysis of several markers of skin differentiation (loricrin, filaggrin and involucrin) showed reduced levels of these proteins in *p23*^*-/-*^ skin (Fig 1D), indicating that the differentiation process is perturbed in embryonic skin in the absence of p23. Skin thinning could be partially due to a reduced number of cells, either because of increased apoptosis or altered proliferation or some combination of both. In fact, p23 has previously been linked to caspase activation and apoptosis. p23 can be C-terminally truncated by caspases 3, 7 and 8, and this is enhanced by geldanamycin [37], and it was reported that p23 cleavage and degradation can be induced by apoptotic stimuli [38]; the effects if the proposed p23 inhibitor gedunin are enhanced by caspase 7 cleavage of p23 [39]. In light of these findings, we decided to check the extent of spontaneous apoptosis in *p23*^*-/-*^ skin. As shown in Fig 1E, TUNEL staining in *p23*^*-/-*^ versus wild-type skin revealed that there is a significant increase in the number of apoptotic cells in *p23*^*-/-*^ skin. Proliferation is affected as well, albeit not as one might have expected based on the dramatically reduced skin thickness: *in vivo* BrdU staining revealed a significantly increased proliferation rate in *p23*^*-/-*^ skin (Fig 1F), which may be substantially counterbalanced by the dramatically increased apoptosis.

### Absence of p23 phenocopies some defects of GR-deficient skin

**Fig 2:**
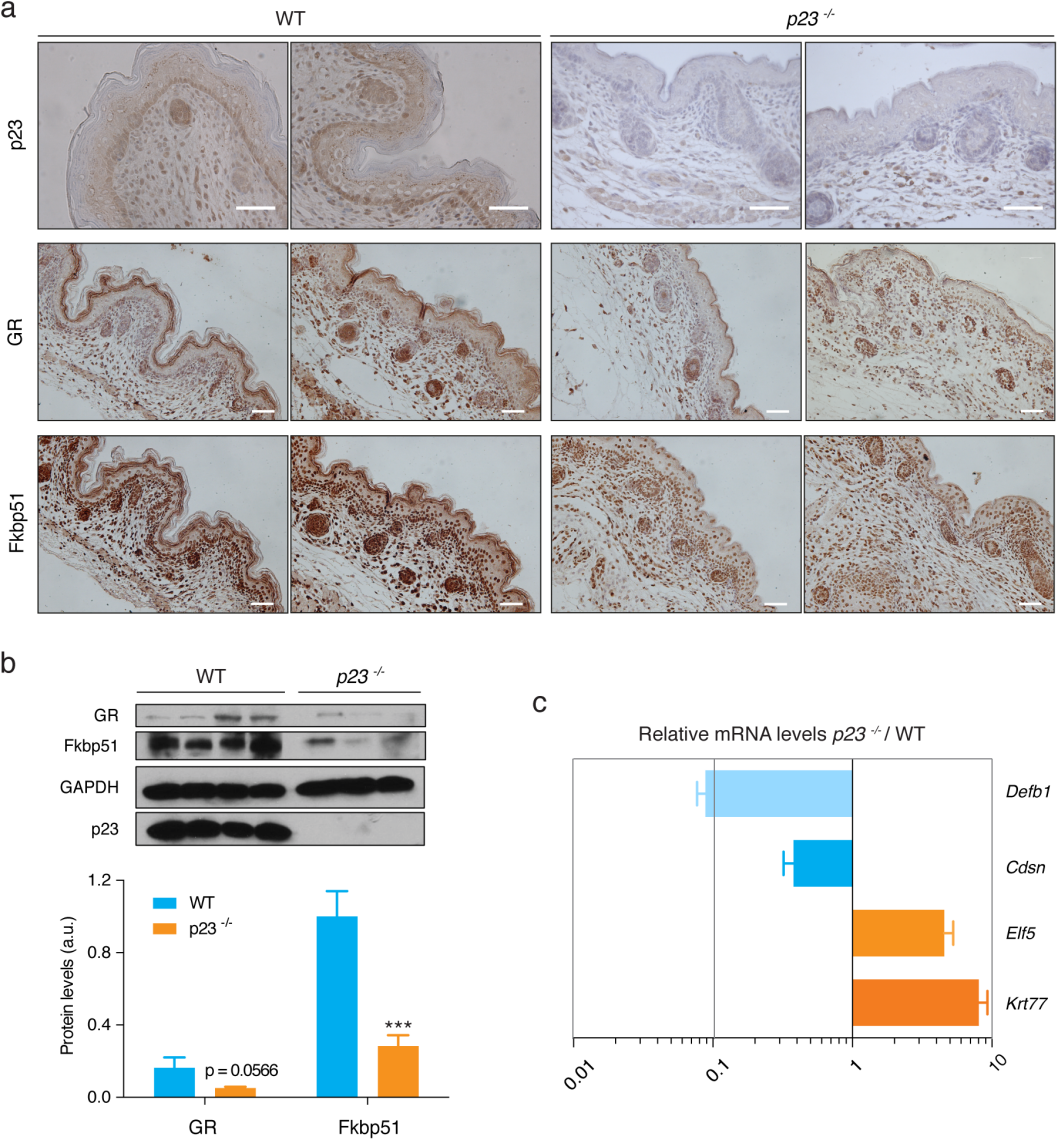
Expression of p23, GR, Fkbp51 and other GR target genes in E18.5 skin. (**a**) Immunohistochemical staining for p23, GR, and Fkbp51 in E18.5 skin sections. Note that GR staining was detected almost exclusively in basal keratinocytes, with very few positive cells in the suprabasal layers, and that GR is also present in the dermal compartment. Fkb51 levels were markedly lower in the epidermis of *p23*^*-/-*^ E18.5 embryos, while expression of GR was slightly lower in some but not all *p23*^*-/-*^ skin sections compared to wild-type. For each protein and genotype, sections of two different embryos are shown. White bars indicate 50 μm. (**b**) Representative immunoblot (top panel) of extracts from 4 WT and 3 *p23*^*-/-*^ embryos shows reduced GR protein levels in E18.5 epidermis in some but not all *p23*^*-/-*^ E18.5 epidermal samples compared to wild-type. The quantitation of two immunoblots with a total of 6 WT and 7 *p23*^*-/-*^ samples is shown as a bar graph. A trend to a reduction of GR levels is suggested by the statistical analysis (p = 0.0566), whereas Fkbp51 levels are undoubtedly decreased in *p23*^*-/-*^ E18.5 epidermis (*** p<0.001). Immunoblotting for p23 was used to confirm the genotype and GAPDH served as loading control. (**c**) Relative mRNA levels of several other GR target genes in *p23*^*-/-*^ *vs*. wild-type E18.5 epidermis, quantified by qPCR. Results are representative of three independent experiments, each with the 6 WT and 7 *p23*^*-/-*^ samples.

In order to define the types of skin cells where p23 might act, we determined the pattern of p23 protein expression in mouse embryonic skin by immunohistochemical staining (Fig 2A; top panel). This revealed expression of p23 in hair follicles, basal keratinocytes, some cells of the stratum spinosum and stratum granulosum and also cells of the dermis. Considering the role of p23 as an Hsp90 co-chaperone and the striking parallels of the phenotype of the *p23*^*-/-*^ mouse with the phenotype of the mouse lacking the Hsp90 client GR, we wanted to clarify if the lack of p23 has an effect on the levels of GR in embryonic skin. As can be seen in the immunohistochemical analysis of Fig 2A (middle panel) and the immunoblot of Fig 2B, the levels of GR protein do not unambiguously correlate with a specific genotype. This result is in agreement with our previous findings with mouse embryonic fibroblasts, in which levels of GR also did not always correlate with the presence or absence of p23 [22]. Overall, GR levels nevertheless appear to be reduced in the absence of p23. Next, we reasoned that GR function may be compromised despite the lack of a clear effect on GR levels. We therefore assessed the expression of a direct GR target gene, *Fkbp51*, at the protein level. In this case, we found significantly lower levels of Fkbp51 in histological sections and by immunoblotting in p23^-/-^ E18.5 skin (Fig 2A and 2B). We used qPCR to look at several other *GR* targets in the skin and noticed significant differences in the mRNA levels of several genes whose expression had previously been linked to perturbed GR function in the skin (Fig 2C) [27,36]. The mRNAs for defensin β-1 (*Defb1*), which is related to the innate immune response and epithelial defense, and corneodesmosin (*Cdsn*), associated with the later stages of epidermal differentiation, are significantly downregulated; the mRNA for E74-like factor 5 (*Elf5*) and keratin-77 (*Krt77*), both normally induced earlier during epidermal development, are upregulated. Thus, the effects in E18.5 *p23*^*-/-*^ skin samples were similar to what had been observed for *GR*^*-/-*^ skin.

### Effects of p23 on keratinocytes are cell-autonomous

**Fig 3:**
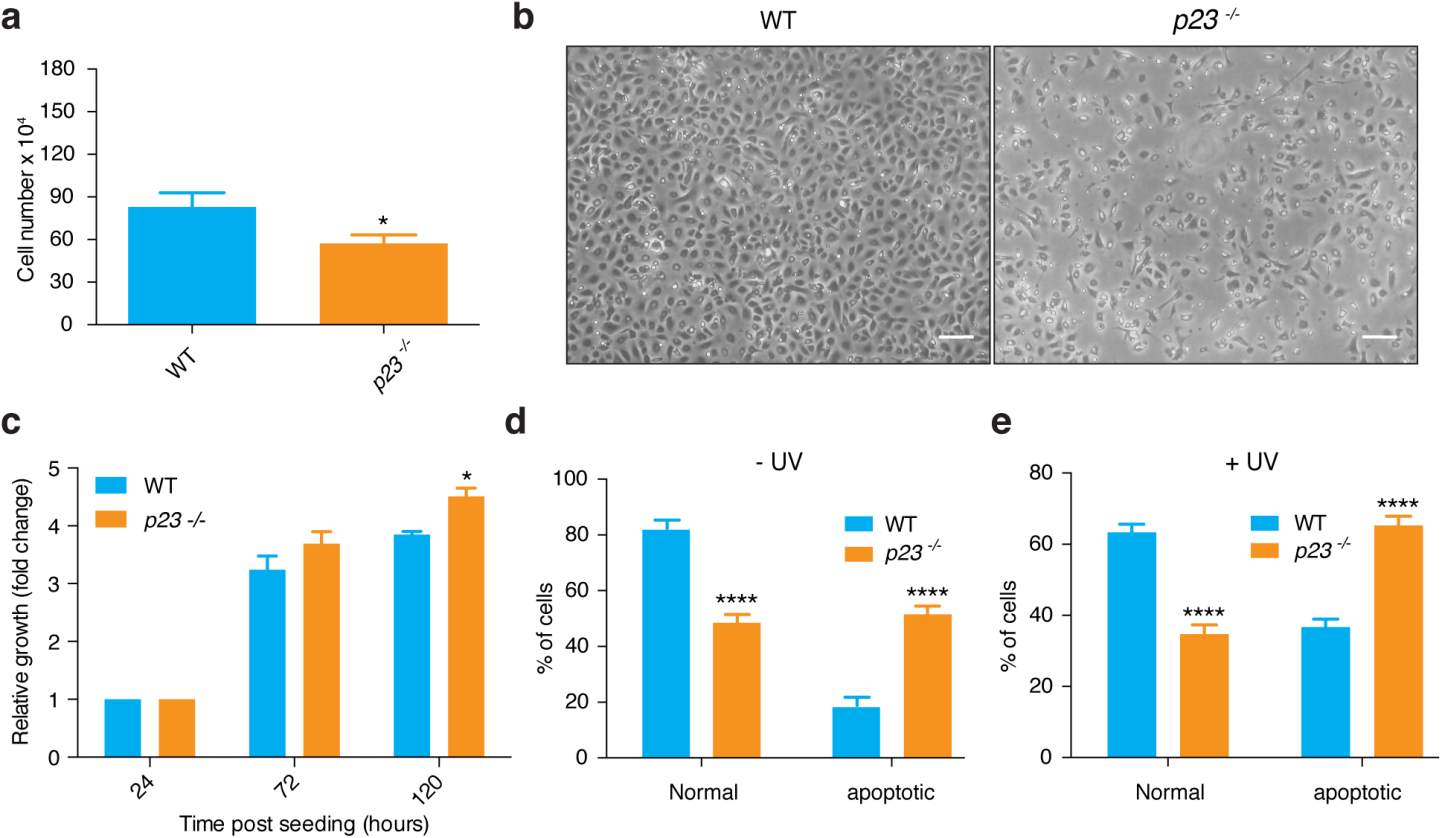
Isolation of primary MPKs. (**a**) Total number of MPKs that can be obtained from the epidermis of individual *p23*^*-/-*^ E18.5 embryos compared to wild-type is significantly lower (* p<0.05; n: WT = 24, *p23*^*-/-*^ = 26). (**b**) Representative micrographs showing differences in cell confluency after seeding MPKs; bars = 50 μm. (**c**) Increased growth rate of *p23*^*-/-*^ MPKs; * p value at 120 h <0.05 (n: WT = 3, *p23*^*-/-*^ = 3). (**d, e**) Increased apoptosis of *p23*^*-/-*^ compared to wild-type MPKs without (**d**; **** p<0.0001; n: WT = 10, *p23*^*-/-*^ = 6) and following UV treatment (**e**; **** p<0.0001; n: WT = 6, *p23*^*-/-*^ = 11).

In order to find out whether the above-mentioned abnormalities in proliferation, differentiation and apoptosis in *p23*^*-/-*^ skin indicate cell-autonomous requirements for p23, we decided to isolate mouse primary epidermal keratinocytes (MPKs) for *in vitro* analyses. Due to the fact that embryonic skin without p23 is significantly thinner than that of the wild-type, we expected to obtain lower yields of isolated MPKs from individual E18.5 embryos, which was indeed the case as can be seen in Fig 3A and 3B. Note that since the isolation of MPKs can be technically quite challenging, we isolated MPKs from wild-type and *p23*^*-/-*^ embryos in parallel and based our analysis on a rather large number of embryos. Moreover, since the BrdU staining had revealed hyperproliferation in *p23*^*-/-*^ skin samples, we compared cell numbers of wild-type and *p23*^*-/-*^ MPKs at 24, 72 and 120 h after seeding the exact same number of cells; we found that there were significantly more *p23*^*-/-*^ than wild-type MPKs at the 120 h time point (Fig 3C). Interestingly, this is reminiscent of the observation that *GR*^*-/-*^ keratinocytes display a hyperproliferative phenotype [26]. Furthermore, we wanted to confirm whether the phenotype of increased apoptosis in *p23*^*-/-*^ skin was also recapitulated with *p23*^*-/-*^ MPKs *in vitro*. For this purpose, we measured annexin V staining by flow cytometry without and with prior UV treatment of the cells; MPKs without p23 proved to have increased spontaneous as well as UV-induced apoptosis (Fig 3D and 3E).

**Fig 4:**
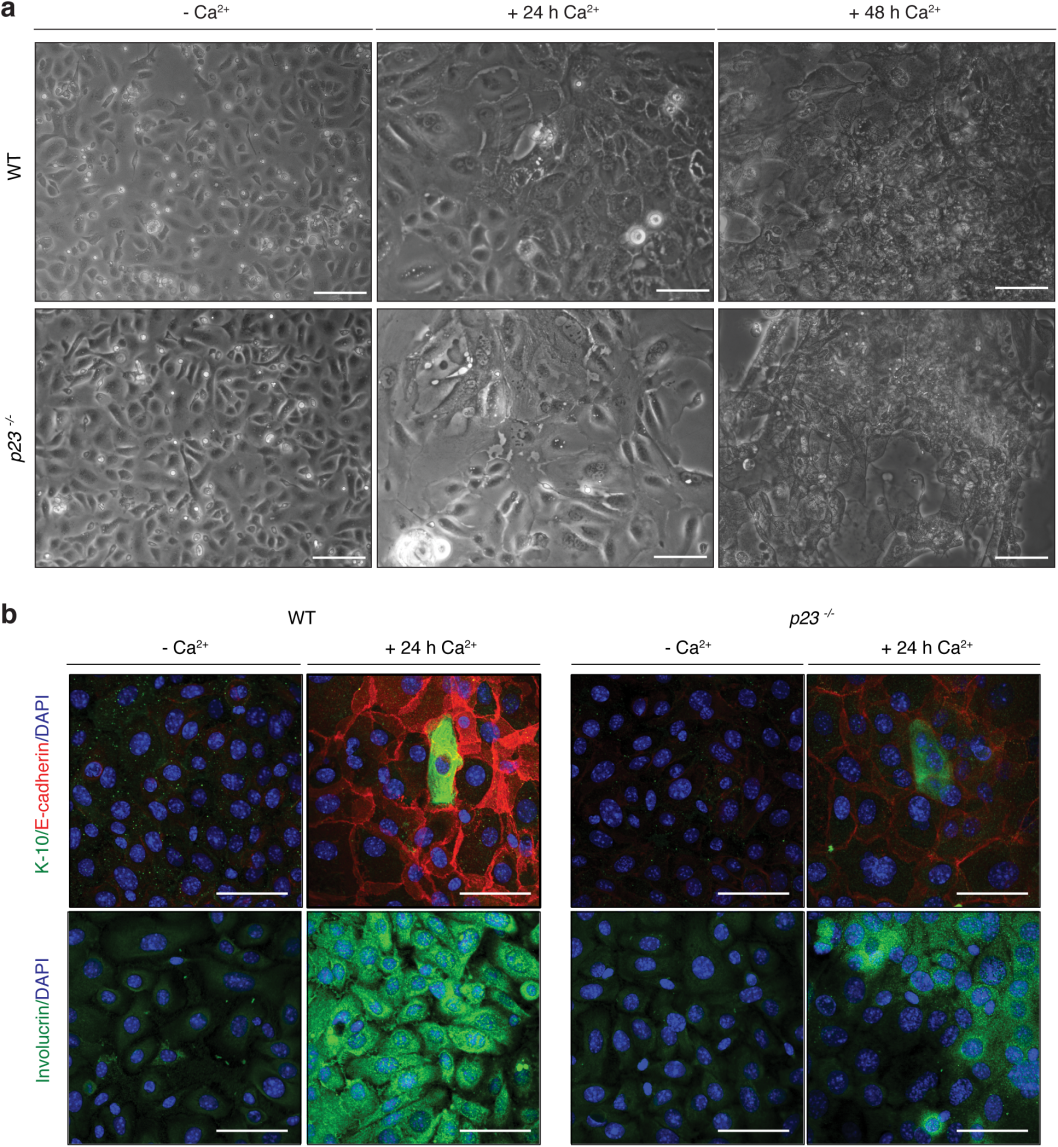
Differentiation of *p23*^*-/-*^ MPKs *in vitro* is impaired. (**a**) Undifferentiated wild-type and *p23*^*-/-*^ MPKs from E18.5 embryos were induced to differentiate with 1.2 mM Ca^2+^ for 24 h and 48 h. No obvious morphological differences were noted by phase contrast microscopy (bars = 50 μm). (**b**) Immunofluorescence staining for epithelial markers demonstrates impaired differentiation of *p23*^*-/-*^ MPKs. Note that only a subset of cells are expected to express K-10 at the relatively early 24 h time point. K-10, keratin-10.

Keratinocyte differentiation can be induced *in vitro* by adding calcium [40]. We used this treatment to assess the ability of *p23*^*-/-*^ MPKs to differentiate. As can be seen in Fig 4A, *p23*^*-/-*^ cells did not show any obvious morphological differences relative to wild-type 24 h or even 48 h after addition of 1.2 mM Ca^2+^. To investigate this in more detail, we performed immunofluorescence staining for markers of early (keratin-10 and E-cadherin) and late (involucrin) differentiation (Fig 4B). We found that *p23*^*-/-*^ MPKs show decreased levels of staining for all three, indicating that even though MPKs are able to differentiate in the absence of p23, differentiation is impaired. The reduced E-cadherin expression is likely to have a negative impact on cell-cell contacts and on the integrity of the epithelial barrier function, consistent with our original findings with whole embryos [22].

### Some effects of glucocorticoids on MPKs are influenced by the p23 status

**Fig 5:**
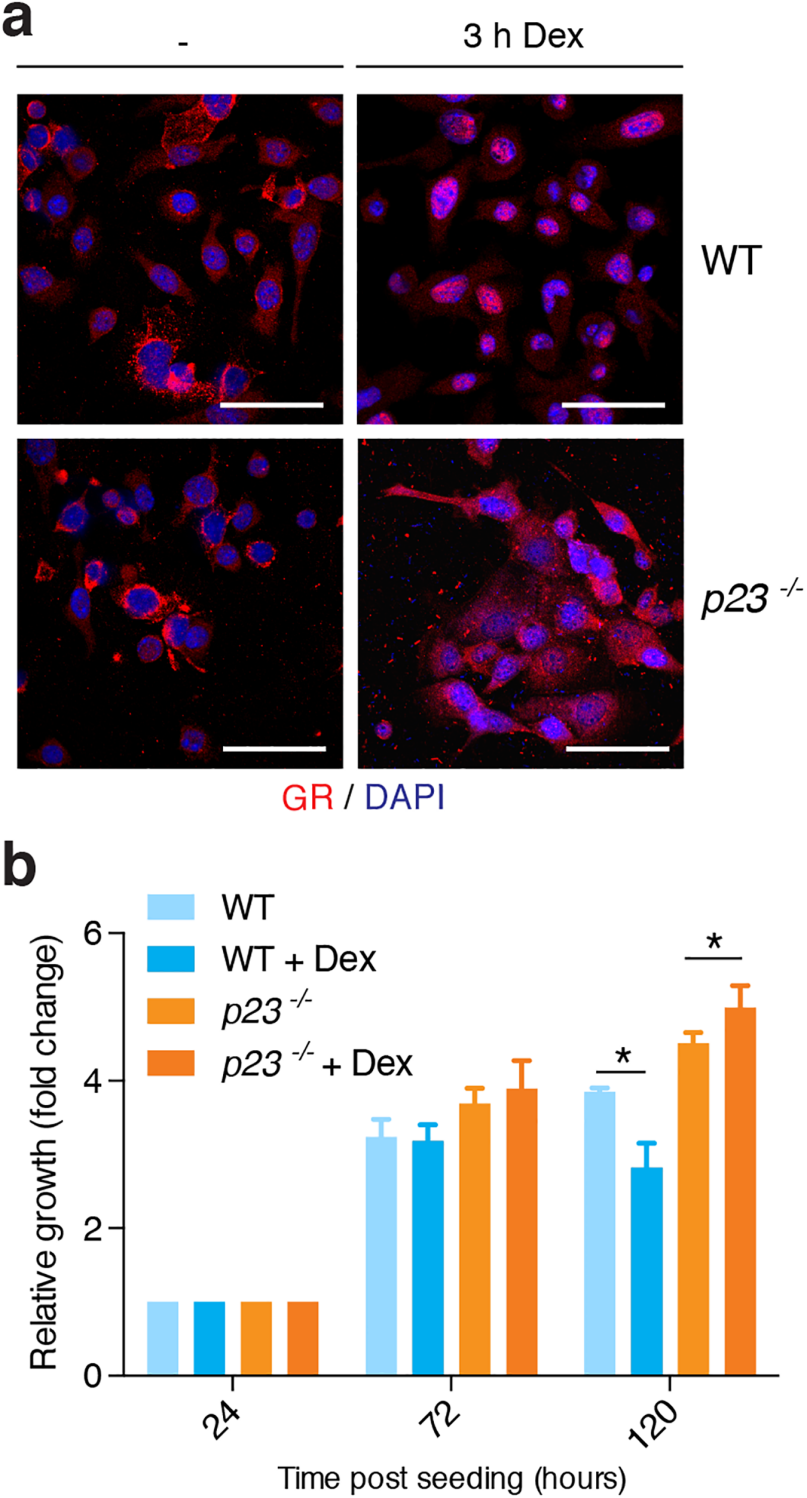
Dex differentially affects *p23*^*-/-*^ versus wild-type MPKs. (**a**) Nuclear localization of GR is impaired in *p23*^*-/-*^ MPKs. Immunofluorescence micrographs of cells treated with 100 nM Dex for 3 h and stained with an antibody to GR (bars = 50 μm). (**b**) Opposite effects of 1 μM Dex on the proliferation of wild-type (* p<0.05) and *p23*^*-/-*^ (* p<0.05) MPKs 120 h after seeding (n: WT = 3, *p23*^*-/-*^ = 3).

Considering the connections between p23 and GR, we set out to investigate the influence of p23 on the response of isolated MPKs to glucocorticoids. The activation of GR can be monitored by assessing its hormone-induced nuclear localization [41]. To investigate the Dex-induced nuclear localization of endogenous GR in *p23*^*-/-*^ MPKs, the cells were treated with 100 nM Dex for 3 hours and, as expected, the majority of GR in wild-type cells within this time accumulated in the nucleus. In contrast, in *p23*^*-/-*^ cells, we observed a reduced nuclear localization under the same conditions (Fig 5A), confirming our previous results with exogenously expressed GR [22]. These results are also consistent with our observation that GR target genes in skin are misregulated (see above). When we analyzed the impact of Dex on the proliferation of MPKs, the effects of the addition of the synthetic glucocorticoid dexamethasone (Dex) turned out to depend on the genotype. At 120 h after seeding in the presence of Dex, the number of wild-type MPKs was reduced whereas Dex had a modest growth stimulatory effect on *p23*^*-/-*^ MPKs (Fig 5B). It is conceivable that the effects on proliferation are due to the direct or indirect dysregulation of certain genes as suggested by the data shown in Fig 2B and 2C.

**Fig 6:**
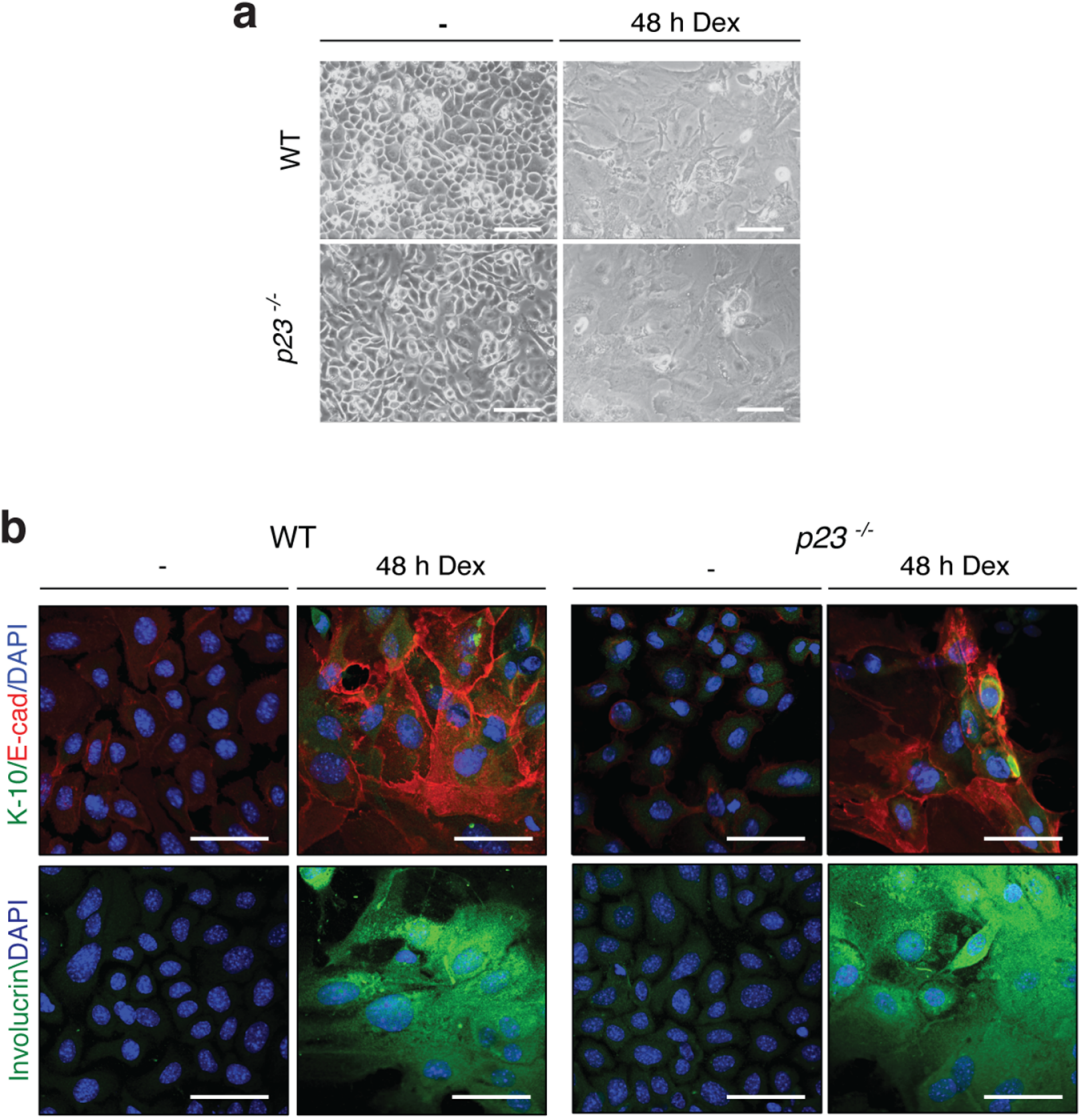
GR-mediated effects on MPK differentiation are not affected by loss of p23. (**a**) Phase contrast micrographs of MPKs induced to differentiate with 1 μM Dex for 48 h. (**b**) Immunofluorescent staining for the differentiation markers keratin-10 (K-10), E-cadherin and involucrin in wild-type and *p23*^*-/-*^ MPKs treated with 1 μM Dex for 48 h (bars = 50 μm).

In contrast to these results indicating defects in GR maturation and signaling, we found that the Dex-stimulated differentiation is not impaired, at least not in isolated MPKs. It has been shown that addition of Dex to wild-type MPKs induces differentiation over time [26]; although we could see morphological changes in response to Dex, there was no obvious difference between the two genotypes (Fig 6A). Unlike Ca^2+^, which failed to fully induce differentiation of MPKs devoid of p23 (Fig 4B), Dex was able to induce full differentiation irrespective of genotype, as judged by immunofluorescence staining for differentiation markers (Fig 6B). Although a relatively high concentration of Dex (1 μM) was used here, 100 nM gave similar results (data not shown).

## Discussion

In our initial report on the phenotype of the *p23* gene disruption, we highlighted the prominent skin phenotype; we speculated based on the resemblance to the phenotype of the GR knockout and on experiments with mouse embryo fibroblasts that it may, at least in part, be due to defective GR maturation and signaling [22]. Our characterization had remained incomplete and the fact that p23 was missing from all cells did not allow us to conclude that p23 is required in the skin itself or any particular cell type of the skin. Here we have further characterized the skin phenotype, and we could demonstrate with isolated MPKs that some defects are due to keratinocyte-specific functions of p23.

At the phenotypic level, we confirm the striking resemblance of the *p23* gene disruption mutant with *GR* knockout mice [25–28]. The epidermis is considerably thinner with an almost absent stratum corneum. There are fewer hair follicles, skin differentiation markers are reduced, there are more apoptotic cells and signs of hyperproliferation. Apart from the superficial similarities between *p23* and *GR* mutant embryos, we found that in the absence of p23, GR target genes are misregulated in embryonic skin, and that glucocorticoid signaling is partially defective in isolated embryonic MPKs. GR cannot be fully activated, and the impact of the synthetic glucocorticoid Dex on the proliferation of MPKs is opposite when comparing wild-type and p23-null MPKs.

Although we may speculate that GR is a major target of the p23 or p23-Hsp90 system in the skin, we cannot conclude that it is the only one. It is likely that some defects of p23-null skin relate to GR- and perhaps even Hsp90-independent functions of p23 [42–44]. These have yet to be characterized and it is impossible to guess which ones may be relevant to the skin phenotype reported here. Specifically, we do not yet know whether the increased spontaneous or UV-stimulated apoptosis and impaired Ca^2+^-induced differentiation of p23-null MPKs are linked to a GR defect. In fact, GR-null MPKs differ from p23-null MPKs in that they can be induced to differentiate properly with Ca^2+^ [26] suggesting that this response may require a GR-independent function of p23. Surprisingly, we found that induction of differentiation by Dex does not appear to be affected by the absence of p23. One complication is that Dex also activates the mineralocorticoid receptor (MR) albeit with reduced affinity and efficiency [45]. MR in the epidermis does play an important role, mediates some glucocorticoid effects and is required along with GR for proper keratinocyte differentiation [46]. While MR, just like GR, is itself an Hsp90 client [47], its p23 dependence is less well understood. Overexpression of p23 has been reported to have opposite effects on GR and MR transcriptional activities in both mammalian cells and yeast, stimulating GR and inhibiting MR [16]. More recently, a differential requirement for Hsp90 itself was suggested by the finding that HDAC6-reversible Hsp90 acetylation affects the interaction of Hsp90 with MR and the subcellular localization of apo-MR, but not its transcriptional response whereas GR activity is severely compromised [48]. How this relates to the ability of p23 to promote the transfer of immature GR from Hsp70 to Hsp90 *in vitro* [49], and whether MR would behave differently, is not known. Clearly, further investigations will be necessary to dissect the relative contributions of GR and MR to skin development and differentiation, and to understand their relative dependence on the Hsp90 molecular chaperone machine in general, and on p23 in particular. Interpreting the p23 skin phenotype in terms of an Hsp90 defect is not straightforward either. The Hsp90β gene knockout is an early embryonic lethal [50] and the Hsp90α gene knockout, albeit viable, does not lead to any obvious skin phenotype [51]. Secreted Hsp90α plays an important role in skin wound healing as a response to hypoxia [52–54], but p23 is not thought to be associated with secreted Hsp90.

We see keratinocyte-autonomous defects in the absence of p23, but we cannot exclude that the loss of p23 functions in other cell types of the epidermis or even in deeper layers of the skin contribute to the skin phenotype. Similarly, the postnatal functions of p23 in the mouse, in any tissue, remain unknown. If GR is indeed one of the key targets of p23, one could postulate that p23 plays a variety of postnatal functions beyond the skin, as does GR [55]. Our efforts to develop a conditionally mutated *p23* allele have not yet been successful. However, this is definitely the way to go to explore the p23-GR connection in more detail and to uncover additional functions of p23 that may in many cases be overshadowed by the flailing GR.

Although not much is known about the regulation of p23 expression and protein stability, there are some precedents that are worth mentioning. Apoptotic stimuli and stress of the endoplasmic reticulum can lead to the caspase-mediated cleavage of p23 [37,38,56]. p23 levels may be upregulated in certain types of cancer [57,58] and by ischemia [59], and downregulated in atherosclerotic plaques [60]. Thus, it is conceivable that certain physiological or pathological influences could modulate p23 levels and thereby affect skin integrity even in the adult.

## Materials and methods

### p23 mutant mouse strain and processing of embryos

The p23 mutant mouse strain and the PCR-based genotyping with DNA isolated from mouse tail biopsies have previously been described; all of the work described here was performed with mouse line A [22], generated from a gene trap insertion into the *p23* gene *Ptges3* of clone W069F07 (see http://www.genetrap.org). Mice were maintained as heterozygotes and housed in standard conditions. E18.5 embryos were collected at 18.5 d after conception. Dorsal skin excised from E18.5 embryos was either rapidly frozen in liquid nitrogen for protein isolation, fixed in 4% paraformaldehyde in phosphate buffered saline (PBS) for histological analyses or immunohistochemistry, or processed for preparation of MPKs as described below. Animals to be sacrificed were euthanized in a chamber with carbon dioxide. Animal research was done according to Swiss law and with approval of local (the "Commission cantonale pour les expériences sur animaux" and the "Office vétérinaire cantonal") and federal authorities ("Office vétérinaire fédéral").

### Histology and immunohistochemistry

E18.5 dorsal skin samples destined for histological analyses were processed, paraffin embedded and sectioned prior to H&E or immunohistochemical staining according to standard protocols. Primary antibodies against p23, filagrin and involucrin were diluted 1:1'000, the one against GR 1:100, for Fkbp51 1:400, and for loricrin 1:2'000; secondary antibodies were diluted 1:2'000.

### Morphometric analyses: Epidermal thickness and hair follicle number

Representative pictures of H&E stained skin sections were randomly chosen and the epidermal width or number of hair follicles within five individual fields of view was calculated for each section using the software ImageJ. Experiments were performed in at least three individuals of each genotype.

### TUNEL staining

In order to identify apoptotic cells in skin sections DeadEnd Fluorometric TUNEL System (Promega) was used according to manufacturer’s instructions. Experiments were performed with at least three individuals of each genotype.

### *In vivo* epidermal BrdU staining

Intraperitoneal injection of BrdU at 130 μg/g of mouse body weight (Roche) was done 1 h before sacrifice in order to detect epithelial cell proliferation. To determine BrdU incorporation immunohistochemistry of paraffin-embedded skin sections using a mouse anti-BrdU monoclonal antibody (Roche) was performed. Total number of cells along with BrdU-positive cells was counted per 200 μm of interfollicular epithelium in each section. Experiments were performed with at least three individuals of each genotype.

### Antibodies

Antibodies against keratin-10 (K-10; PRB-159P; Covance), E-cadherin (H-108; Santa Cruz), involucrin (SY5; Abcam), GR (M-20 or H-300; Santa Cruz Biotechnology), p23 (JJ3; a gift from David O. Toft, Mayo Clinic), Fkbp51 (HI51B; a gift from Marc Cox, University of Texas at El Paso), loricrin, (PRB-145P; Covance), filaggrin (PRB-417P; Covance) and GAPDH (Abcam) were used for immunohistochemistry, immunofluorescence or immunoblotting. Secondary biotin-conjugated (Vector Laboratories), peroxidase-conjugated (DAKO) or FITC- or Texas Red- (Vector Laboratories) conjugated anti-rabbit or anti-mouse antibodies were used for immunohistochemistry, immunoblotting or immunofluorescence, respectively.

### Immunoblotting

Protein extracts from the epidermis of E18.5 embryos were prepared according to standard protocol. Equal amounts of protein extracts were subjected to SDS-PAGE and followed by standard immunoblotting procedures. Dilutions of primary antibodies against p23, GR, Fkbp51, and GAPDH were 1:1'000, 1:500, 1:1'000, and 1:10'000, respectively. Experiments were performed with samples from at least three individuals of each genotype. Quantification of protein levels was done using ImageJ software.

### Harvesting of MPKs

Embryos were placed in betadine (Mundipharma), 70% ethanol, no calcium Dulbecco's PBS without calcium (DPBS) (from Gibco), 70% ethanol and DPBS for 5 min each. The tail of each embryo was then collected for genotyping, the limbs and snout were cut off and an incision was made from the posterior part of the back up to the snout before the skin was carefully peeled off and washed in DPBS. Skins were placed epidermal side up in dispase II (3 U/ml in medium; Roche) in separate 35 mm culture dishes and incubated overnight at 4°C. Epidermis was then separated from the dermis with forceps and placed in new dishes containing 1 ml of accutase (CELLnTEC) for 20 min at room temperature. Afterwards 2 ml of low calcium CnT-07 Epidermal Keratinocyte medium (CELLnTEC) and 2 ml of DPBS was added to each dish and repeated pipetting was used to dissociate keratinocytes from the epidermal sheet. Dissociated cells were then collected, centrifuged for 5 min at 1000 rpm and resuspended in 1 ml of CnT-07 medium. The number of cells obtained from each embryo was calculated using haemocytometer and a specific number of cells was seeded in culture dishes coated with collagen I (Sigma) and immediately used for experiments as indicated. MPKs were cultured in CnT-07 medium at 37°C.

### MPK treatments

To induce keratinocyte differentiation, MPKs were grown until confluency in CnT-07 low calcium medium and then 1.2 mM Ca^2+^ was added for either 24 h or 48 h. Prior to addition of 1 μM Dex or vehicle (ethanol), confluent MPKs were incubated overnight in charcoal-stripped low calcium medium (DMEM without phenol red from Gibco with 4% serum depleted of steroids with charcoal, supplemented with 0.05 mM Ca^2+^ ["low calcium"], 10 ng/ml EGF and antibiotics). Dex was added either at 100 nM for 3 h to induce the nuclear localization of GR or at 1 μM to monitor the effects on proliferation and for 48 h to induce keratinocyte differentiation.

### Immunofluorescence staining

Cells were grown on glass coverslips in 12- or 24-well plates and fixed with methanol:acetone (70:30, v/v). The air-dried coverslips were stored at -20°C until needed or used immediately for immunofluorescence staining. Staining was done by incubation with the primary and secondary antibodies in 1% BSA for 2 h each. Dilutions of primary antibodies against GR, K-10, E-cadherin, and involucrin were 1:200. Coverslips were mounted with DAPI-containing Vectashield mounting medium (Vector Laboratories).

### Annexin V staining

Where indicated, 35 mm culture dishes with confluent MPKs were subjected ultraviolet irradiation at 150 mJcm^−2^ using the UV Stratalinker 1800 (Stratagene), and incubated in fresh medium for 6 h at 37°C. Cells were detached using 0.25% Trypsin-EDTA (Gibco), collected by centrifugation, washed twice in cold PBS and resuspended in 500 μl of 1x binding buffer (0.1 M Hepes pH 7.4, 1.4 M NaCl, 25 mM CaCl_2_). 100 μl of the cell-containing binding buffer solution was transferred to a 5 ml FACS tube, 5 μl of FITC Annexin V (BD Biosciences) and 10 μl of propidium iodide (PI) (50 μg/ml; Invitrogen) were added, cells were gently shaken and incubated for 15 min at room temperature in the dark. Annexin V and PI binding was analyzed by flow cytometry using a BD FACSCanto instrument with the BD CellQuestPro software (BD Biosciences). FITC signal detector (FL1) and PI signal detector (FL3) were used to distinguish between apoptotic and live cells. At least 10'000 events were counted per run.

### MTT cell proliferation assay

MPKs were plated at equal densities in a 96-well plate and their proliferation was monitored by performing MTT assays with 3-(4,5-Dimethylthiazol-2-yl)-2,5-diphenyltetrazolium bromide (Applichem).

### Quantitative real-time PCR

Total RNA was isolated from E18.5 epidermis using guanidinium-acid-phenol as previously described [61]. 1 μg of total RNA was reverse transcribed using random primers (hexadeoxynucleotide mix, Promega) with the GoScript reverse transcriptase (Promega) as indicated by the manufacturer. Expression of GR target genes in the epidermis and of the internal standard GAPDH using specific primers was then analyzed by quantitative real-time PCR with the GoTaq Green Master Mix (Promega) according to the manufacturer’s instructions. The forward and reverse primers for *Krt77* were CCAGGTGCTACAGACAAAATGG and GCTGACTGATGAACTCCTCGAA, for *Elf5*, ACCGATCTGTTCAGCAATGAAG and CGCTTGGTCCAGTATTCAGG, for *Cdsn*, TTGCTGATGGCCGGTCTTATT and GCCAGTCTTTCCAATGAGACAAG, for *Defb1*, CCTCATCTGTCAGCCCAACT and GTGAGAATGCCAACACCTGG, and for *Gapdh*, GCACAACAGGAAGAGAGAGACC and AGGGGAGATTCAGTGTGGTG.

### Statistical analyses

GraphPad Prism (GraphPad Software) was used for statistical analyses. Results were subjected to Student’s t-test analysis. Graphs represent arithmetic means ± standard error of the mean (SEM). Differences with P values of P<0.05 were considered to be statistically significant.
